# Effectiveness of gabapentinoids in orthopedic surgeries: a systematic review and meta-analysis of postoperative pain, and opioid-sparing effects

**DOI:** 10.1186/s12871-025-03291-9

**Published:** 2025-11-14

**Authors:** Fatimah Abdulaziz Almuqad, Ahmed Adel Almuhammady, Ghaida Talea Alsaedi, Joud Adel Osailan, Sulafah Ibrahim Alalawi, Yousef Abdullah Rahmah Allah, Ahmed Osailan

**Affiliations:** 1 College of Medicine, Al-Rayan Colleges, Madinah, Saudi Arabia; 2 College of Medicine, Taibah University, Madinah, Saudi Arabia; 3 Anesthesia Department, College of Medicine, Taibah University, Madinah, Saudi Arabia

**Keywords:** Gabapentinoids, Postoperative pain, Orthopedic surgery, Opioid sparing

## Abstract

**Background:**

Gabapentinoids, including gabapentin and pregabalin, are increasingly used in perioperative settings to manage postoperative pain. Despite their widespread use, the clinical evidence regarding the treatment of gabapentinoids in terms of improvement of pain, reduction of opioid consumption and the enhancement of functional recovery is inconclusive. This systematic review and meta-analysis aimed at evaluating the efficacy of gabapentinoids in orthopedic surgeries, with a focus on their role as standalone interventions without the influence of other analgesic modalities like spinal anesthesia or nerve blocks.

**Methods:**

We conducted a search of electronic databases, including PubMed, Scopus, and Web of Science, to identify randomized controlled trials (RCTs) to identify randomized controlled trials (RCTs) evaluating the use of gabapentinoids in orthopedic surgeries. Fourteen RCTs which met the inclusion criteria were included in the meta-analysis mean differences (WMDs) and risk ratios (RRs) with 95% confidence intervals (CIs) were calculated for continuous and binary outcomes, respectively. Data on postoperative pain reduction, opioid-sparing effects, and adverse events were extracted and analyzed using a random-effects model. Weighted mean differences (WMDs) and risk ratios (RRs) with 95% confidence intervals (CIs) were calculated for continuous and binary outcomes, respectively. Heterogeneity was assessed using the I^2^ statistic.

**Results:**

The meta-analysis revealed that gabapentinoids significantly reduced postoperative pain intensity at 24 hours (WMD: -0.57). Although statistically significant, the mean reductions in the mean reductions in pain intensity did not exceed the minimal clinically important difference (MCID) of 1.0 point on a 0–10 numeric rating scale, indicating limited clinical relevance. Gabapentinoids were also followed by a lower risk of nausea (RR: 0.68) but an increased likelihood of dizziness (RR: 1.25).

Heterogeneity was high in the category of pain (I^2^ >50%), which suggested variability in the study designs and the involved patients.

**Conclusion:**

While gabapentinoids demonstrate statistically significant benefits by reducing post-operative pain and opioid use in orthopedic surgeries, their clinical effect is still limited.

**Supplementary Information:**

The online version contains supplementary material available at 10.1186/s12871-025-03291-9.

## Introduction

The class of medications known as gabapentinoids, which include gabapentin and pregabalin, was first introduced as anticonvulsants in the 1990 s and later approved to treat various chronic neuropathic pain disorders [1]. They have since then found widespread use in perioperative medicine, specifically in managing acute postoperative pain [2]. Pregabalin and gabapentin act through a variety of pharmacological pathways, such as blocking Ca^2^⁺ transmission through high-voltage gated channels and competing with L-amino acid transporters. Gabapentinoids reduce neurotransmitter release and synaptic excitability, ultimately leading to neuronal suppression and decreased pain transmission [3]. The aforementioned process enables them to effectively eliminate the pain signals, in particular in cases of neuralgia and postoperative pain [4].

Medical authorities recently expressed serious concerns regarding the net clinical benefit of gabapentinoids as well as their side effects, such as respiratory depression and misuse risk [1]. However, studies on the effects of pregabalin and gabapentin on acute postoperative pain in recent years have shown that they can help avoid acute nociceptive pain following surgery. Both medications can reduce the need for opioids, lower the opioid dosage, and minimize the side effects associated with opioid use. They also have the potential to reduce the intensity of initial postoperative pain. Additionally, they might help alleviate persistent postoperative pain and reduce preoperative anxiety [5, 6].

According to a randomized controlled trial by Omara et al., giving patients undergoing orthopedic surgery 150 mg of pregabalin orally one hour prior to spinal anesthesia was successful in prolonging the initial request for postoperative analgesics and reducing postoperative discomfort. Significantly, it improved sleep quality post-operative and a longer duration of sensory and motor block [7]. On the other hand, a lot of other studies have been able to testify of these conclusions which demonstrate that pregabalin truly affects the pain level for orthopedic surgery patients when comparing placebo to defend it [1, 3]. These data give an indication that if used in the perioperative period, gabapentinoids may be very influential and may play a significant role in the highly painful and long recovery surgeries.

Orthopedic procedures often involve both nociceptive and neuropathic pain components due to bony manipulation and periosteal nerve injury. Gabapentinoids, which target neuropathic mechanisms, may therefore confer unique benefits in this population [5, 6]. Despite the growing body of evidence, data specifically assessing the effects of gabapentinoids on postoperative pain, and opioid-sparing effects in orthopedic surgeries without the influence of other analgesic modalities, such as spinal anesthesia or nerve blocks, are still lacking. Therefore, this systematic review and meta-analysis aim to critically evaluate the available evidence to determine the effectiveness of gabapentinoids as a standalone intervention in enhancing these outcomes among orthopedic surgery patients.

## Methods

This systemic review and meta-analysis was performed in accordance with the PRISMA 2020 (Preferred Reporting Items for Systematic Reviews and Meta-Analysis) guidelines [8] and was registered in PROSPERO (CRD420251003335).

### Databases and search strategy

The following databases were searched: PubMed, Scopus, and Web of Science to identify relevant studies published up to February 10, 2025. Key search terms used are a combination of: ("Gabapentin"OR"Pregabalin"OR"calcium channel blocker"OR"voltage-gated calcium channel inhibitor") AND ("Orthopedic Procedures"OR"Orthopedic Surgery"OR"Musculoskeletal Surgery") AND ("Postoperative Pain"OR"Pain"OR"Pain Management"OR"Acute Pain"OR"Chronic Pain"OR"Analgesia"OR"Pain Score"OR"VAS Score"OR"NRS Score").

### Inclusion and exclusion criteria

We included studies that involved adult patients (aged 18 years and above) undergoing orthopedic surgeries, where gabapentin or pregabalin was administered preoperatively, intraoperatively, or postoperatively. Eligible studies compared these interventions to placebo or standard care and reported outcomes such as postoperative pain reduction (measured by visual analogue scale [VAS] or numerical rating scale [NRS]), opioid-sparing effects, functional recovery (e.g., range of motion or early ambulation), or the incidence of chronic postoperative pain. Only randomized controlled trials (RCTs) published in English within the last 15 years were considered.

Studies were excluded if they evaluated gabapentin or pregabalin for purposes unrelated to postoperative pain management, lacked a placebo or standard care comparison group, did not report at least one of the predefined outcomes, or were non-randomized. Additionally, we excluded studies published in non-English languages, those without full-text availability, and duplicate publications. Since the inclusion criteria already specified adult orthopedic populations, we have not repeated the exclusion of pediatric or non-orthopedic studies, in line with the reviewer’s suggestion to avoid redundancy.

### Data extraction

The data extraction was conducted by 7 independent reviewers who initially screened the titles and abstracts of all retrieved articles to identify potentially relevant studies. After that, a full text articles screening was performed for the selected studies following our inclusion and exclusion criteria. And then any discrepancies among the reviewers were resolved by discussion with another reviewer. A standardized data extraction form was developed using Microsoft Excel to ensure accuracy. The extracted key elements include study characteristics (Authors, year of publication, country, study design) and participant details (sample size, age, surgery type, preexisting conditions, baseline pain score), intervention details (type of drug used, dosage, route of administration, timing, number of doses,) control group details (type of control, administration details and timing) and the outcomes of interest (postoperative pain reduction at 6,12,24,48,and 72 hours in addition to subacute and chronic pain relief, adverse effects and the main findings). To aid interpretation of clinical relevance, a predefined threshold for the minimally clinically important difference (MCID) was applied. Based on prior literature, an MCID of 1.0 point on a 0–10 numeric rating scale (NRS) for pain was considered the minimum clinically important change [9]. There is no widely accepted MCID for opioid consumption, but a 30% reduction from baseline has been suggested in prior studies as a meaningful threshold and was used for reference [9].

### Risk of bias assessment

To assess the risk of bias in the included studies, we used the JBI Critical Appraisal Tool for Randomized Controlled Trials (RCTs), to evaluate 13 domains such as randomization, allocation concealment, blinding, baseline similarity, and outcome measurement [10]. Two independent reviewers conducted the assessment, resolving disagreements through discussion or consultation with a third reviewer. Two independent reviewers performed the assessment, resolving disagreements through discussion or consultation with a third reviewer. The studies were classified as low, moderate, or high risk of bias based on the number of domains with potential bias. The main points of emphasis were whether participants and providers were blinded, whether outcomes were measured reliably, and whether follow-up was complete.

### Data synthesis and statistical analysis

We used the MetaXL tool for statistical analysis to create and compile forest plots as well as aggregate data from 14 randomized control trials (RCTs). Different outcomes like the reduction of postoperative pain were analyzed by weighted mean differences (WMDs) using 95% confidence intervals (CIs) for continuous outcomes, while the binary outcomes such as the adverse effects like nausea, headache, drowsiness, visual disturbances, and urinary retention, were tested using the risk ratios (RRs) with 95% CIs. The I^2^ statistic was the key method for determining the level of heterogeneity across the studies, and it generated the following pattern of results according to the values: 25%, 25–50%, and 50%-showed low, moderate, and large discrepancies, respectively. The sample variability was later considered as we employed the random effects model for our analysis, as it provides a more conservative estimate by considering both within-study and between-study variability. The forest plots were utilized in overall effects visualizations, and statistical significance was tested at *p* < 0.05.

## Results

### Study selection process

**Fig. 1:**
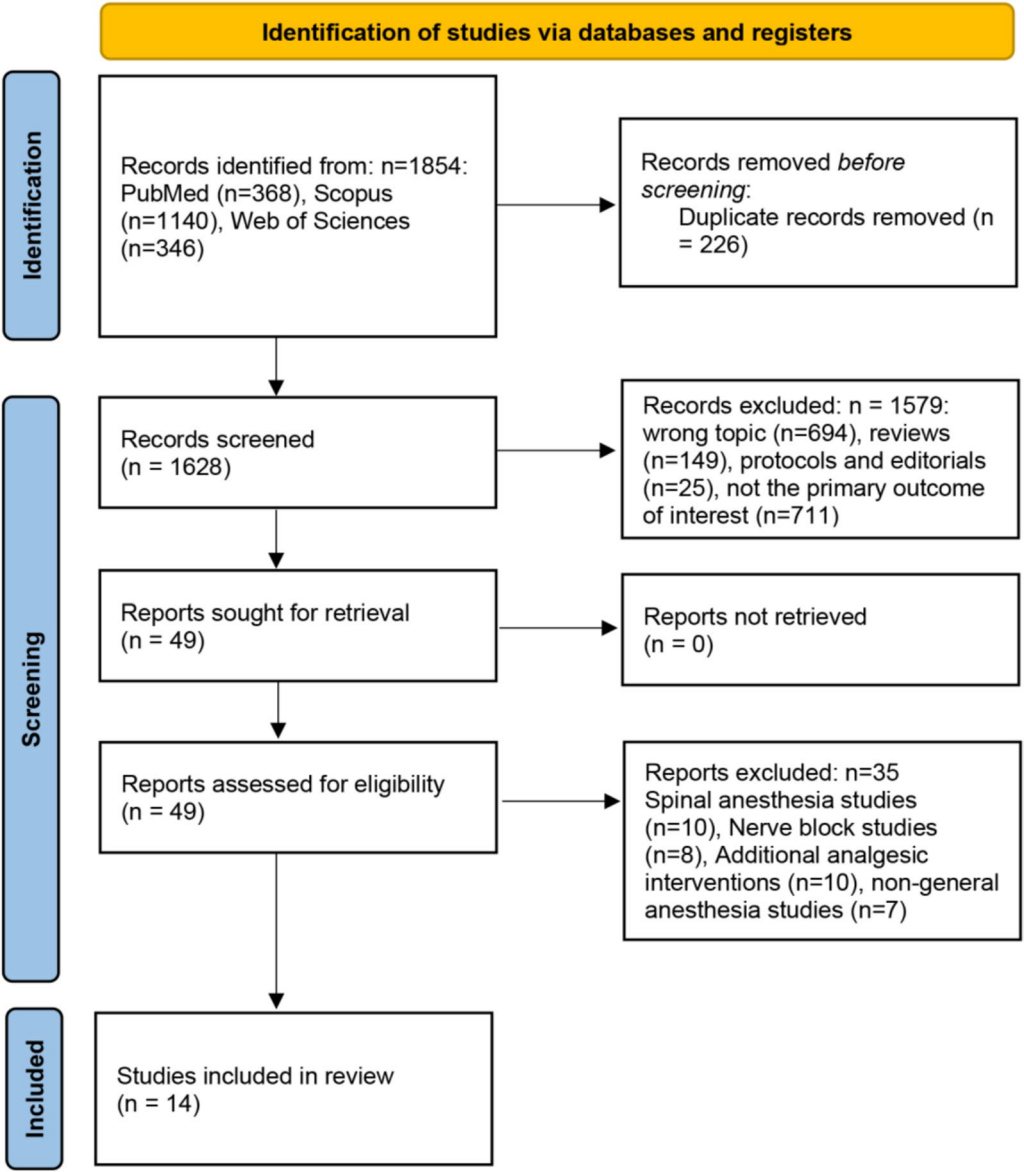
PRISMA flowchart summarizing the selection process for studies included in the systematic review and meta-analysis

The PRISMA flow diagram (Fig. 1) outlines the study selection process for this systematic review. A total of 1,854 records were identified from a variety of databases such as PubMed (*n* = 368), Scopus (*n* = 1,140), and Web of Sciences (*n* = 346). After removing 226 duplicate records, 1,628 records were screened. Of these, 1,579 records were excluded for reasons such as wrong topic (*n* = 694), reviews (*n* = 149), protocols and editorials (*n* = 25), and not addressing the primary outcome of interest (*n* = 711). Following this, 49 full-text reports were assessed for eligibility. After excluding 35 studies—due to the use of spinal anesthesia (*n* = 10), nerve blocks

(*n* = 8), additional analgesic interventions (*n* = 10), or non-general anesthesia (*n* = 7), 14 studies were included in the final review [6, 11–23].

### Pain reduction after 12 and 24 Hours

**Fig. 2:**
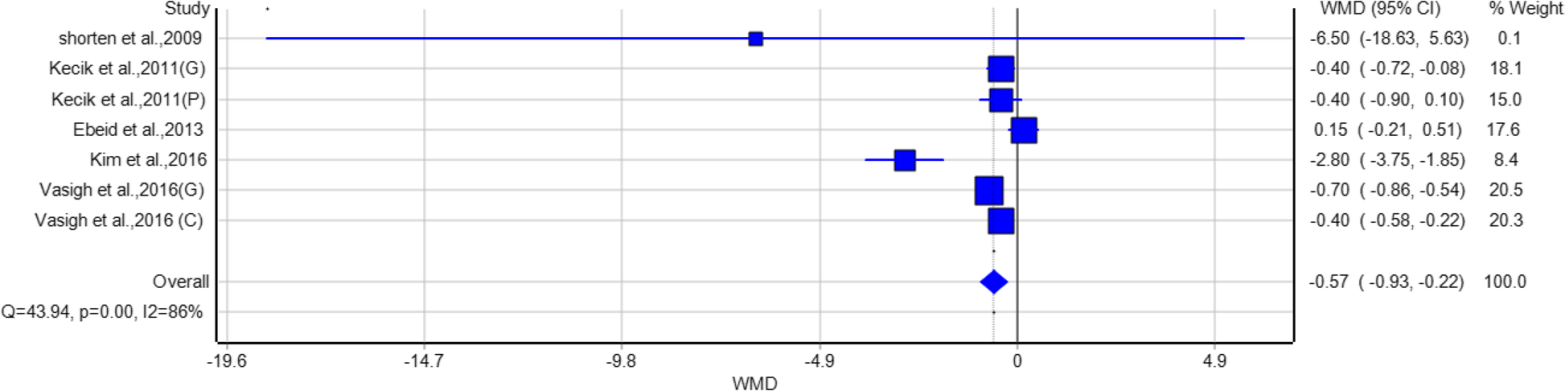
Forest plot demonstrating the effect of gabapentin/pregabalin versus placebo on postoperative pain reduction at 24 hours

The forest plot for pain reduction at 12 hours post-surgery (Fig. 2) showed a pooled Weighted Mean Difference (WMD) of –0.18 (95% CI: –0.83 to 0.46), indicating no statistically significant difference between the intervention and placebo groups. This effect size did not meet the predefined Minimal Clinically Important Difference (MCID) of 1.0, suggesting that any observed reduction in pain was not clinically meaningful. Individual studies varied, with some reporting significant reductions (e.g., Vasigh et al., 2016 [22]; WMD = –1.3, 95% CI: –1.57 to –1.03) and others reporting increases (e.g., Nouri et al. 2024 [6]; WMD = 1.41, 95% CI: 0.95 to 1.87).

**Fig. 3:**
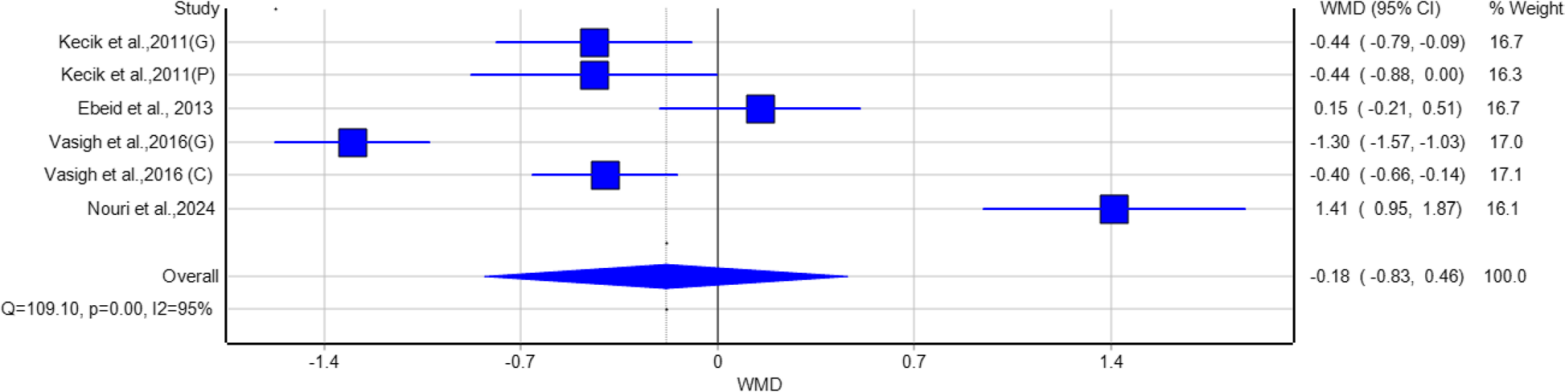
Forest plot demonstrating the effect of gabapentin/pregabalin versus placebo on postoperative pain reduction at 12 hours

For pain reduction at 24 hours post-surgery (Fig. 3), the pooled WMD was –0.57 (95% CI: –0.93 to –0.22), which was statistically significant. However, this value still fell below the predefined MCID of 1.0, suggesting that the reduction in pain, although statistically significant, was likely not clinically meaningful for most patients. Considerable heterogeneity was observed (I^2^ = 86%, *p* < 0.01).

### Adverse effects of gabapentin and pregabalin

**Fig. 4:**
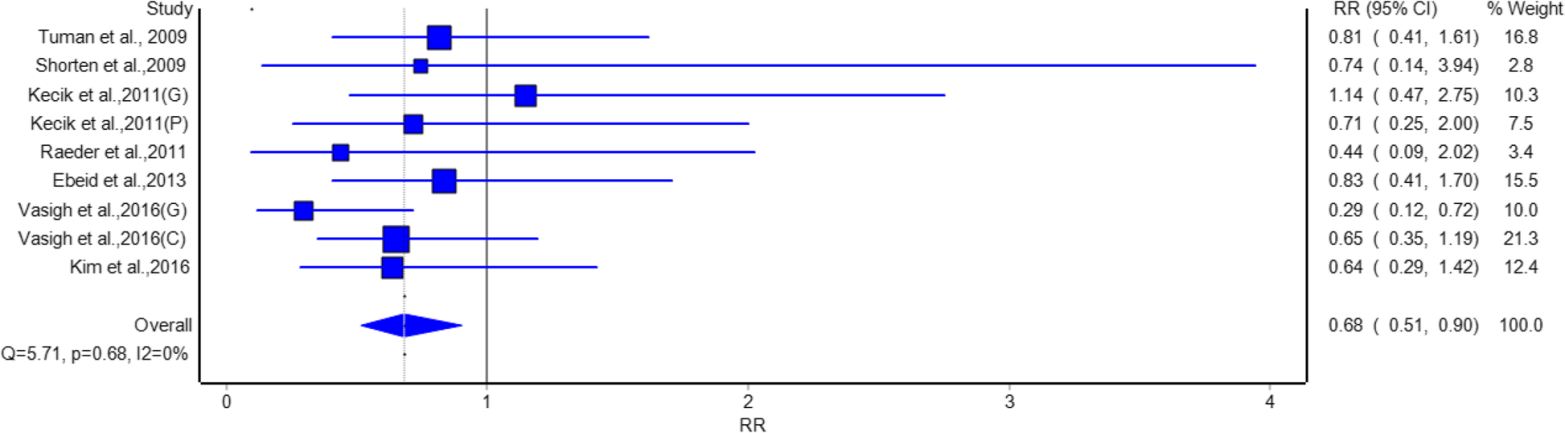
Forest plot showing a comparison of the risk of postoperative nausea between gabapentinoids and placebo groups

The adverse effect analysis reported inconsistent findings regarding the various outcomes. Gabapentin and pregabalin were associated with a statistically significant reduction in the risk of nausea compared to placebo, as shown in Fig. 4. The pooled Risk Ratio (RR) was 0.68 (95% CI: 0.51, 0.90), indicating a 32% lower risk of nausea in the intervention groups. Vasigh et al. 2016 [22] (Gabapentin) (RR = 0.29, 95% CI: 0.12, 0.72) and Yorukoglu et al. [15] (Pregabalin) (RR = 0.71, 95% CI: 0.25, 2.00) studies were the basis of this finding. No heterogeneity was observed (I^2^ = 0%, *p* = 0.88), thus it is a uniform result of the studies.

**Fig. 5:**
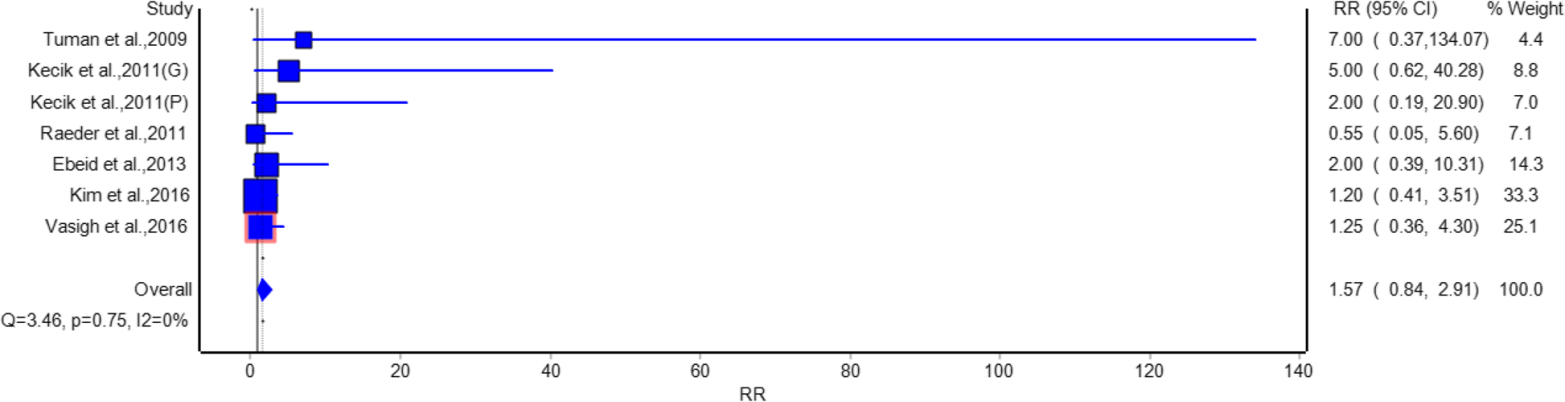
Forest plot showing a comparison of the risk of postoperative headache between gabapentinoids and placebo groups

**Fig. 6:**
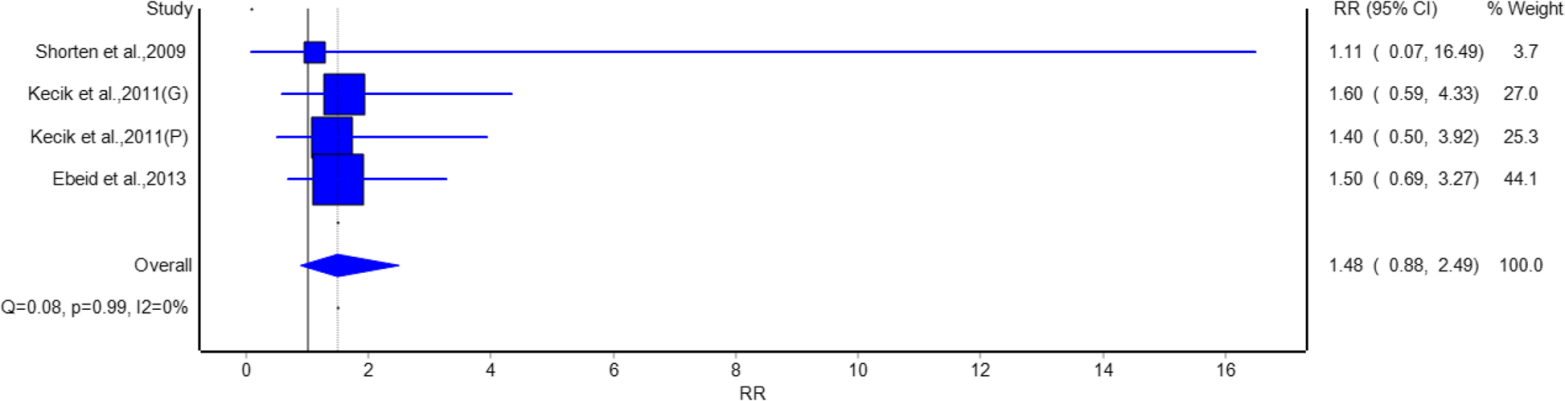
Forest plot showing a comparison of the risk of postoperative somnolence between gabapentinoids and placebo groups

For headache, as shown in Fig. 5, no significant difference in the risk of headache was observed among the intervention and placebo groups. The pooled RR was 1.57 (95% CI: 0.84, 2.91), which means that gabapentin/pregabalin did not have an effect on the risk of headache, because it did not increase or decrease it. Studies such as Ahn et al. 2016 [20] (RR = 1.2, 95% CI: 0.41, 3.51) and Vasigh et al. 2016 [22] (RR = 1.25, 95% CI: 0.36, 4.30) showed no significant effects. No heterogeneity was observed (I^2^ = 0%, *p* = 0.75). The risk of somnolence, as shown in Fig. 6, did not differ significantly between the intervention and placebo groups. The pooled RR was 1.48 (95% CI: 0.88, 2.49), suggesting that gabapentin/pregabalin did not significantly increase the risk of somnolence. Studies such as Yorukoglu et al. [15] (Gabapentin) (RR = 1.6, 95% CI: 0.59, 4.33) and Eskandar et al. [17] (RR = 1.5, 95% CI: 0.69, 3.27) contributed to this finding. No heterogeneity was observed (I^2^ = 0%, *p* = 0.99).

**Fig. 7:**
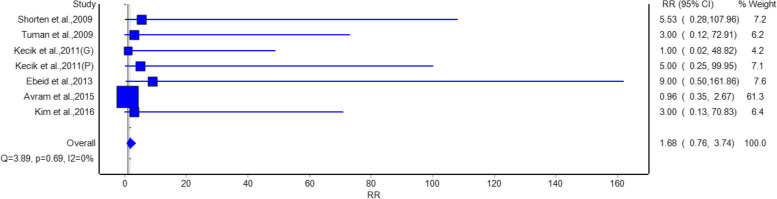
Forest plot showing a comparison of the risk of postoperative visual disturbances between gabapentinoids and placebo groups

Figure 7 shows that there was no significant difference in risk for visual disturbance between the groups that got the intervention and those that got the placebo. The pooled RR was 1.68 (95% CI: 0.76, 3.74), indicating that gabapentin/pregabalin did not significantly increase the risk of visual disturbance. Studies such as Eskandar et al. [17] (RR = 9, 95% CI: 0.50, 161.86) and Paul et al. 2015 [19] (RR = 0.96, 95% CI: 0.35, 2.67) showed no significant effects. No heterogeneity was observed (I^2^ = 0%, *p* = 0.69).

**Fig. 8:**
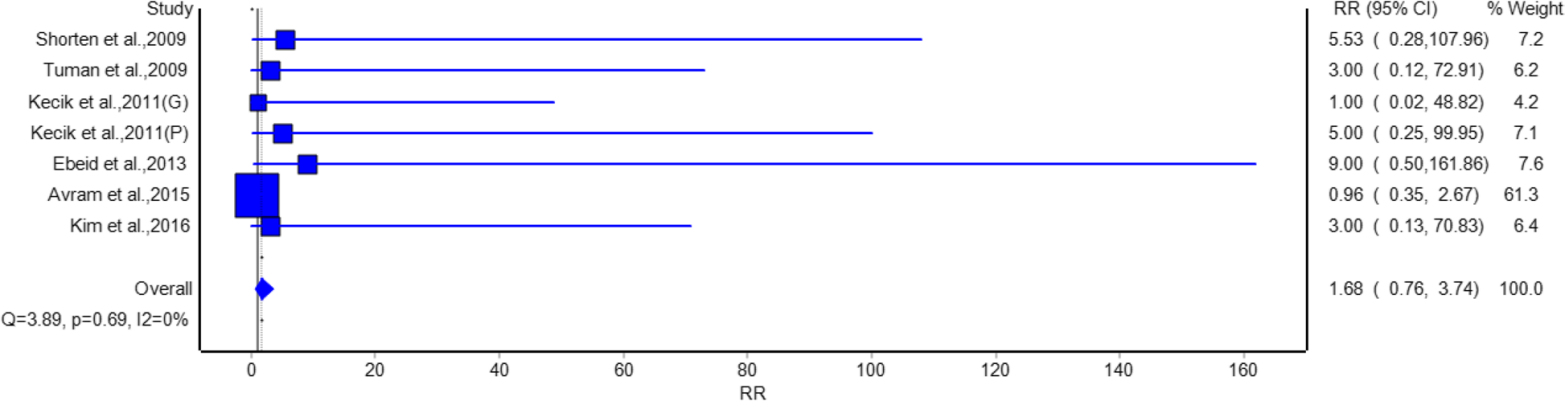
Forest plot showing a comparison of the risk of postoperative urinary retention between gabapentinoids and placebo groups

Even gabapentin and pregabalin studies showed the efficacy of bladder control by exhibiting a least statistically significant reduction in risk of urine retention compared to placebo, as shown in Fig. 8. The RR of 0.63 (95% CI: 0.43, 0.94) was the total, with an intervention group having 37 % fewer occurrence of urine retention. Studies such as Yorukoglu et al. [15] (Gabapentin) (RR = 0.44, 95% CI: 0.15, 1.29) and Vasigh et al. 2016 [22] (Gabapentin) (RR = 0.56, 95% CI: 0.21, 1.50) contributed to this finding. No heterogeneity was observed (I^2^ = 0%, *p* = 0.68) (Tables 1 and 2).

**Table 1:** Characteristics of included studies

**Study ID**	**Country**	**Study design**	**Sample size**	**Population (groups)**	**Patient age (mean ± sd)**	**Surgery type**	**Drug used**	**Dosage (mg/day)**	**Timing of administration**	**Control group**
Burke et al. 2010 [10]	Ireland	Randomized, Placebo-controlled, Double-blind	40	Pregabalin vs. Placebo	Placebo: 41 ± 12.4; Pregabalin: 37 ± 7.8	Lumbar Discectomy	Pregabalin	600	Pre- and post-operative	Placebo (sucrose)
Buvanendran et al. 2010 [11]	USA	Randomized, Placebo-controlled, Double-blind	240	Pregabalin vs. Placebo	Pregabalin: 64.0 ± 8.3; Placebo: 63.3 ± 8.9	Total Knee Arthroplasty	Pregabalin	300–100	Pre- and post-operative	Placebo
Hegarty et al. 2011 [13]	Ireland	Randomized, Placebo-controlled	32	Pregabalin vs. Placebo	Placebo: 41.8 ± 8.1; Pregabalin: 38.8 ± 7.9	Lumbar Discectomy	Pregabalin	300	Pre-operative	Placebo
Khan et al. 2011 [14]	Iran	Randomized, Placebo-controlled	175	Gabapentin (600, 900, 1200 mg) vs. Placebo	40.4–43.6 years	Lumbar Laminectomy	Gabapentin	600–1200	Pre- and post-incision	Placebo
Kim et al. 2011 [15]	South Korea	Randomized, Placebo-controlled, Double-blind	84	Pregabalin (75 mg, 150 mg) vs. Placebo	Placebo: 39.67 ± 11.11; Pregabalin: 44–48	Lumbar Spinal Fusion	Pregabalin	75–150	Pre- and post-operative	Placebo
Yorukoglu et al. 2011 [15]	Turkey	Randomized, Placebo-controlled, Double-blind	90	Gabapentin (1200 mg) vs. Pregabalin (300 mg) vs. Placebo	48.6–51.9 years	Lumbar Laminectomy/Discectomy	Gabapentin/Pregabalin	1200/300	Pre- and post-operative	Placebo
Spreng et al. 2011 [17]	Norway	Randomized, Placebo-controlled,	46	Pregabalin vs. Placebo	Pregabalin: 44.1 ± 10.8; Placebo: 42.9 ± 7.6	Lumbar Discectomy	Pregabalin	150	Pre-operative	Placebo
Eskandar et al. 2013 [18]	Egypt	Randomized, Placebo-controlled,	80	Pregabalin vs. Placebo	Placebo: 42.15 ± 13.08; Pregabalin: 41.3 ± 14.7	Shoulder Arthroscopy	Pregabalin	600	Pre-operative	Placebo
Lunn et al. 2015 [18]	Denmark	Randomized, Placebo-controlled, Double-blind	300	Gabapentin (900 mg, 1300 mg) vs. Placebo	67–70 years	Total Knee Arthroplasty	Gabapentin	900–1300	Pre- and post-operative	Placebo
Paul et al. 2015 [19]	Canada	Randomized, Placebo-controlled, Double-blind	102	Gabapentin vs. Placebo	Gabapentin: 60.9 ± 9.1; Placebo: 60.5 ± 8.5	Total Hip Arthroplasty	Gabapentin	600–200	Pre- and post-operative	Placebo
Ahn et al. 2016 [20]	South Korea	Randomized, Placebo-controlled,	60	Pregabalin vs. Placebo	Pregabalin: 55 ± 9; Placebo: 51 ± 12	Arthroscopic Shoulder Surgery	Pregabalin	150	Pre-operative	Placebo
Vasigh et al. 2016 [22]	Iran	Randomized, Placebo-controlled, Double-blind	114	Gabapentin vs. Celecoxib vs. Placebo	49.5–50.2 years	Laminectomy	Gabapentin	900	Pre- and post-operative	Placebo
Khalili et al. 2017 [23]	Iran	Randomized, Placebo-controlled, Double-blind	96	Gabapentin vs. Paracetamol vs. Placebo	Gabapentin: 41.32 ± 10.83; Paracetamol: 41.08 ± 9.89	Tibial Fracture Surgery	Gabapentin	300	Pre-operative	Placebo
Nouri et al. 2024 [6]	Iran	Randomized, Placebo-controlled, Double-blind	80	Gabapentin vs. Pregabalin	Gabapentin: 41.32; Pregabalin: 41.08	Orthopedic Surgery (Upper Limb)	Gabapentin/Pregabalin	300/150	Pre-operative	Control group (Gabapentin)

**Table 2:** Outcomes of included studies

**Study ID**	**Pain reduction**				**Subacute/Chronic pain reduction**		**Adverse events**	**Main findings**
	**6h**	**12h**	**24h**	**48h**	**4–12 Weeks**	**>3 Months**		
Burke et al. 2010 [10]	-	-	VAS at rest: Pregabalin 17.3 mm vs. Placebo 23.8 mm (P=0.16)	-	PPI-VAS: Pregabalin 37.6 mm vs. Placebo 25.3 mm (*P* = 0.08)	VAS at rest: Pregabalin 10.7 mm vs. Placebo 15.4 mm (*P* = 0.249)	Visual disturbances, somnolence, dizziness (Pregabalin); nausea, vomiting, dizziness (Placebo)	Pregabalin improved pain, function, and quality of life at 3 months post-surgery.
Buvanendran et al. 2010 [11]	-	-	-	-	Neuropathic pain: Pregabalin 0% vs. Placebo 8.7% (*P* = 0.001)	Neuropathic pain: Pregabalin 0% vs. Placebo 5.2% (*P* = 0.014)	Sedation, confusion, dizziness (Pregabalin); similar but lower rates in Placebo	Pregabalin reduced neuropathic pain incidence at 3 and 6 months post-TKA.
Hegarty et al. 2011 [13]	-	C group: 3; PG group: 1	C group: 2; PG group: 1	-	-	-	Nausea, somnolence, dizziness (higher in Pregabalin group)	Pregabalin reduced morphine consumption but did not significantly reduce pain intensity.
Khan et al. 2011 [14]	-	MD: 0.5–1.4 (vs. Placebo)	MD: 0.3–0.7 (vs. Placebo)	-	-	-	Nausea, vomiting, drowsiness, dizziness (higher in Gabapentin 1200 mg group)	Gabapentin 900–1200 mg reduced post-operative pain and morphine consumption.
Kim et al. 2011 [15]	-	-	-	-	-	-	Sedation, headache, dizziness (similar across groups)	Pregabalin 150 mg reduced post-operative pain and opioid consumption.
Yorukoglu et al. 2011 [15]	Placebo: 3.33; Gabapentin: 2.4; Pregabalin: 2.36	Placebo: 2; Gabapentin: 1.56; Pregabalin: 1.56	Placebo: 1.5; Gabapentin: 1.1; Pregabalin: 1.1	-	-	-	Nausea, vomiting, dizziness, somnolence (higher in Gabapentin and Pregabalin)	Both Gabapentin and Pregabalin reduced pain and opioid consumption compared to placebo.
Spreng et al. 2011 [17]	-	-	-	-	-	-	PONV, sedation, dizziness (similar across groups)	Pregabalin reduced post-operative pain and morphine consumption without increasing side effects.
Eskandar et al. 2013 [18]	-	Pregabalin: 2.35; Placebo: 2.75	Pregabalin: 2.1; Placebo: 1.95	-	-	-	Nausea, vomiting, dizziness, somnolence (similar across groups)	Pregabalin reduced post-operative pain after shoulder arthroscopy.
Lunn et al. 2015 [18]	-	-	-	-	-	-	Nausea, dizziness, sedation, visual disturbance (higher in Gabapentin high-dose)	Gabapentin did not significantly reduce post-operative pain or opioid consumption.
Paul et al. 2015 [19]	-	-	-	-	-	-	Nausea, vomiting, dizziness, sedation (similar across groups)	Gabapentin did not significantly reduce post-operative pain or opioid consumption.
Ahn et al. 2016 [20]	Pregabalin: 4.7; Placebo: 7.5	-	Pregabalin: 3.3; Placebo: 6.1	Pregabalin: 3.0; Placebo: 4.5	-	-	Nausea, dizziness, sedation (similar across groups)	Pregabalin reduced post-operative pain and opioid consumption after shoulder surgery.
Vasigh et al. 2016 [22]	Gabapentin: 3.4; Celecoxib: 4.4; Placebo: 5.4	Gabapentin: 1.6; Celecoxib: 2.5; Placebo: 2.9	Gabapentin: 0.7; Celecoxib: 1; Placebo: 1.4	-	-	-	Nausea, vomiting, dizziness, drowsiness (higher in Gabapentin group)	Gabapentin reduced post-operative pain and morphine consumption more effectively than Celecoxib and placebo.
Khalili et al. 2017 [23]	Gabapentin: 4.06; Paracetamol: 4.22; Placebo: 5.31	-	-	-	-	-	Mild side effects (headache, nausea)	Gabapentin and Paracetamol reduced post-operative pain compared to placebo.
Nouri et al. 2024 [6]	Gabapentin: 3.65; Pregabalin: 2.3	Gabapentin: 3.41; Pregabalin: 2.0	-	-	-	-	Dizziness, drowsiness, nausea, vomiting (similar across groups)	Pregabalin was more effective than Gabapentin in reducing post-operative pain.

### Quality and risk of bias assessment

The JBI tool's appraisal of risks of bias revealed that there were differences in methodological quality across the included studies (Table 3). The majority of these studies showed low risk while some exhibited moderate risks that were either due to inadequate information on allocation concealment or lack of blinding (participants, providers, or outcome assessors) or small sample sizes. The interventions from Buvanendran et al. [11] and Lunn et al. [18] were robust, as they both were double-blind and had large samples. However, the others like Hegarty et al. [13] and Eskandar et al. [18] did not have blinding details, which led to a performance and detection bias issue.

**Table 3:** Quality assessment of the included studies using the JBI critical appraisal tool

**Study author (year)**	**Q1**	**Q2**	**Q3**	**Q4**	**Q5**	**Q6**	**Q7**	**Q8**	**Q9**	**Q10**	**Q11**	**Q12**	**Q13**	**Overall risk of bias**	**Rationale for key concerns**
Burke et al. 2010 [10]	Yes	Unclear	Yes	Yes	Yes	Yes	Unclear	Yes	Yes	Yes	Yes	Yes	Yes	Moderate	Blinding of outcome assessors (Q7) unclear; small sample size (*n* = 40).
Buvanendran et al. 2010 [11]	Yes	Yes	Yes	Yes	Yes	Yes	Yes	Yes	Yes	Yes	Yes	Yes	Yes	Low	Large sample (*n* = 240), robust design (double-blind), complete follow-up.
Hegarty et al. 2011 [13]	Yes	Unclear	Yes	No	No	Yes	Unclear	Yes	Yes	Unclear	Yes	Yes	Yes	Moderate	Not double-blind (Q4, Q5:"Randomized, Placebo-controlled"only). Blinding of assessors (Q7) unclear.
Khan et al. 2011 [14]	Yes	Unclear	Yes	No	No	Yes	No	Yes	Yes	Unclear	Yes	Yes	Yes	Moderate	No blinding (Q4–Q7: design lacks"double-blind"descriptor). High-dose variability (600–1200 mg).
Kim et al. 2011 [15]	Yes	Yes	Yes	Yes	Yes	Yes	Yes	Yes	Yes	Yes	Yes	Yes	Yes	Low	Double-blind, standardized outcomes.
Yorukoglu et al. 2011 [15]	Yes	Yes	Yes	Yes	Yes	Yes	Yes	Yes	Yes	Yes	Yes	Yes	Yes	Low	Well-reported, three-arm comparison.
Spreng et al. 2011 [17]	Yes	Unclear	Yes	Yes	Yes	Yes	Unclear	Yes	Yes	Yes	Yes	Yes	Yes	Moderate	Blinding of outcome assessors (Q7) unclear. Small sample (*n* = 46).
Eskandar et al. 2013 [18]	Yes	Unclear	Yes	No	No	Yes	No	Yes	Yes	Unclear	Yes	Yes	Yes	Moderate	Not double-blind (design lacks this descriptor). Blinding of assessors (Q7) unclear.
Lunn et al. 2015 [18]	Yes	Yes	Yes	Yes	Yes	Yes	Yes	Yes	Yes	Yes	Yes	Yes	Yes	Low	Large sample (*n* = 300), rigorous design.
Paul et al. 2015 [19]	Yes	Yes	Yes	Yes	Yes	Yes	Yes	Yes	Yes	Yes	Yes	Yes	Yes	Low	Double-blind, balanced groups.
Ahn et al. 2016 [20]	Yes	Unclear	Yes	No	No	Yes	No	Yes	Yes	Yes	Yes	Yes	Yes	Moderate	Not double-blind (design lacks this descriptor). Blinding of assessors (Q7) unclear.
Vasigh et al. 2016 [22]	Yes	Yes	Yes	Yes	Yes	Yes	Yes	Yes	Yes	Yes	Yes	Yes	Yes	Low	Triple-arm design, clear reporting.
Khalili et al. 2017 [23]	Yes	Yes	Yes	Yes	Yes	Yes	Yes	Yes	Yes	Yes	Yes	Yes	Yes	Low	Well-reported, double-blind.
Nouri et al. 2024 [6]	Yes	Unclear	Yes	Yes	Yes	Yes	Unclear	Yes	Yes	Unclear	Yes	Yes	Yes	Moderate	Blinding of assessors (Q7) unclear. Small sample (*n* = 80).

**Notes: Q1**: Was true randomization used for assignment of participants to treatment groups? **Q2**: Was allocation to treatment groups concealed? **Q3**: Were treatment groups similar at the baseline? **Q4**: Were participants blind to treatment assignment? **Q5**: Were those delivering the treatment blind to treatment assignment? **Q6**: Were treatment groups treated identically other than the intervention of interest? **Q7**: Were outcome assessors blind to treatment assignment? **Q8**: Were outcomes measured in the same way for treatment groups? **Q9**: Were outcomes measured in a reliable way? **Q10**: Was follow-up complete, and if not, were differences between groups in terms of their follow-up adequately described and analyzed? **Q11**: Were participants analyzed in the groups to which they were randomized? **Q12**: Was appropriate statistical analysis used? **Q13**: Was the trial design appropriate, and were any deviations from the standard RCT design accounted for in the conduct and analysis of the trial?

## Discussion

The aim of this systematic review and meta-analysis was to assess the effectiveness of gabapentinoids (gabapentin and pregabalin) in treating postoperative pain, opioid-sparing effects as well as functional recovery outcomes in orthopedic surgeries. The analysis showed that gabapentinoids were correlated with a statistically significant reduction in pain at 24 hours after the surgery, with the pooled Weighted Mean Difference (WMD) of 0.57 (95% CI: −0.93, −0.22). It is suggested that gabapentin and pregabalin could minimize postoperative pain in the short run. Nevertheless, the pooled mean difference in postoperative pain scores did not exceed the predefined Minimal Clinically Important Difference (MCID) of 1.0. In addition, there is a big heterogeneity (I^2^ = 86%, *p* < 0.01) in the data which indicates that the size of the effect may be different based on the amount of dosage, time of administration, and the type of the surgical procedure. For instance, a study comparing gabapentin and celecoxib in pain management after laminectomy showed a significant decrease in the pain of patients with gabapentin [22], while others, such as another study that compare the effects of oral gabapentin (GBP) and pregabalin (PGB) in pain control after orthopedic surgery on the upper limb, reported a rise in pain, possibly due to differences in the patients'populations or surgical techniques [6].

At 12 hours post-surgery, the pooled WMD of −0.18 (95% CI: −0.83, 0.46) did not reach statistical significance, suggesting that gabapentinoids may not be as effective in the immediate postoperative period. One possible reason for this might be that the pharmacokinetics of gabapentinoids are such that they need longer periods to achieve plasma levels that are therapeutic [24]. The high heterogeneity (I^2^ = 95%, *p* < 0.01) indicates the necessity of a standardized dosing regimen. It also calls for more research in optimizing the timing of the administration.

In comparison, Verret et al. (2020) found a statistically significant but clinically insignificant reduction in pain intensity at 6-, 12-, 24-, and 48-hours post-surgery, with mean differences ranging from −10 to −3 on a 100-point scale [1]. These findings were similar, suggesting that while gabapentinoids might offer some pain relief, they don't make a big impact that patients can feel. A systematic review looked specifically at people with burn injuries and found that gabapentinoids significantly lowered intense pain soon after treatment. The pain scores dropped noticeably within 24 hours with an MD of −1.06 [25]. This suggests that gabapentinoids might work better for burns, where the cause of pain might be different compared to surgeries on bones or joints. In another study about pain relief after cesarean sections, it was found that a 600 mg dose of gabapentin reduced pain more effectively than a placebo [26].

However, this study did not compare gabapentin directly with more common painkillers like NSAIDs. These studies show that the effectiveness of gabapentinoids depends on the type of surgery and the cause of pain. They might be more useful in certain situations like burns or cesarean sections than in others.

Gabapentinoids, such as gabapentin and pregabalin, have been shown to help in lessening the opioid consumption in several studies. For example, a meta-analysis has found that gabapentin can enhance pain relief from opioids while allowing people to use less of them [27]. In addition to the mentioned one, a study found that when gabapentin was taken in the first 24 hours after surgery, the need for opioids dropped significantly, with a standard mean difference of −1.35 [28]. Besides this, another study showed that most of the patients who received treatment with gabapentin and pregabalin after spine surgery had less pain and the necessity for opioid supplements than a placebo group [29]. This finding is crucial in light of the ongoing opioid crisis, as using less opioids lowers the risk of addiction and negative side effects.

A systematic review studied how effective gabapentinoids are for managing pain after surgery. It also observed a slight reduction in opioid consumption with gabapentinoids, with a mean difference of −7.90 mg of intravenous morphine equivalent at 24 hours post-surgery [1]. However, this reduction was not clinically significant, and the study highlighted the increased risk of adverse events such as dizziness and visual disturbance [1].

Pain linked to orthopedic operations is usually of a complex nature as it involves nociceptive, inflammatory, and neuropathic nature [30]. This is the case because advanced tissue trauma, nerve damage, and the release of inflammation mediators are contributing to the overall complication associated with postoperative pain management [30]. Multifaceted pain's diversity is a clear point of intersection for research into orthopedic surgeries and as such, a unique environment is created for an evaluation of the gabapentinoids'efficacy. According to Verma et al. (2014), pregabalin interferes with voltage-gated Ca^2^⁺ channels, and specifically binds to the alpha-2-delta subunit to induce analgesic effects [31]. It has shown an impressive effectiveness in improvements of neuropathic pain symptoms, e.g., allodynia and hyperalgesia in both preclinical and clinical studies, as a result, it has emerged as the first-line agency for such neuropathic painful (such as the one related to orthopedic operations) [31]. The findings show that gabapentinoids are the medicines that orthopedic surgeons need to handle complex pain profiles coming from their patients.

A review article highlighted that gabapentinoids reduce opioid consumption by 30–50% in neuropathic pain settings, with a number needed to treat (NNT) for ≥30% pain reduction ranging from 4–7 [32]. This indicates moderate opioid-sparing potential, particularly in contexts where neuropathic pain is a significant component, such as in spinal surgeries or joint replacements. However, the extent of the reduction in opioid treatment may not always be clinically significant, and the possible benefits of the therapy must be taken into consideration along with the risks of side effects.

While the reductions in pain scores and opioid use reached statistical significance in some analyses, the magnitude of these effects was limited. Specifically, the pooled mean difference in postoperative pain scores did not exceed the predefined Minimal Clinically Important Difference (MCID) of 1.0. This disconnect between statistical significance and clinical relevance highlights a key limitation in the practical utility of gabapentinoids for postoperative pain management in orthopedic surgery. These findings suggest that, although measurable effects exist, their real-world benefit to patients may be limited.

Analysis of adverse events suggests that gabapentinoids were related to a statistically significant reduction in nausea with relative risk of 0.68 and urine retention relative risk of 0.63 as compared with a placebo group. Hence, gabapentinoids are suggested to have benefits like reducing common postoperative complications beside pain relief. However, no significant differences were observed in the risk of headache, somnolence, or visual disturbance, indicating that gabapentinoids are generally well-tolerated with a favorable safety profile.

A systematic review on burn injuries indicated that gabapentinoids do not statistically increase the risk of urinary retention compared to control groups, which aligns with our findings. However, another study mentioned that gabapentin has been found to lead to urinary incontinence in very few cases, especially with higher doses (600–3600 mg/day) [25]. This implies that while gabapentinoids do not cause urinary retention, they may still provoke other urinary-related side effects, mainly at higher doses [33].

A large observational study examined the risks of serious adverse outcomes associated with gabapentinoid use in UK primary care patients [34]. It found that people using gabapentinoids had higher rates of drug misuse, overdoses, and severe injuries than those who did not use these drugs. Pregabalin users faced a higher risk of overdosing compared to those on gabapentin [34]. The significant risk factors for the formation of adverse outcomes in an individual include younger age, current smoking, history of substance use or mental illnesses, and simultaneous intake of other CNS depressants such as opioids and benzodiazepines. These findings suggest that patients need to be selected cautiously and proper education concerning combining with other CNS depressants needs to be given.

According to the JBI tool, the risk of bias assessment has exhibited methodological quality variation in the studies that were included. Some studies, like those by Buvanendran et al. [11] and Lunn et al. [18], demonstrated low risk of bias due to their strong design and large sample sizes, However, other studies showed moderate risk. This was because they didn’t clearly explain how they chose their participants or didn’t use blinding methods, which meant they didn’t hide who was getting what treatment. These problems could have affected the study results. It’s important to address these issues in future studies to make the findings more trustworthy and useful.

### Clinical implications

While gabapentinoids were associated with statistically significant reductions in postoperative pain and opioid consumption, the magnitude of these effects did not exceed established thresholds for minimal clinically important difference (MCID). Therefore, their overall clinical benefit in orthopedic surgery remains modest. Given the variability in patient response and the potential for adverse effects such as dizziness or somnolence, gabapentinoid use should not be routine but rather individualized. Clinicians should carefully consider patient-specific factors, such as age, comorbidities, and type of surgical procedure, when deciding whether to include these agents in perioperative pain management protocols.

### Limitations

This study has several important limitations. First, the high degree of heterogeneity across the included studies, due to variations in gabapentinoid dosing and timing of administration, means that the overall findings should be interpreted with caution. Subgroup analyses based on dose or type of orthopedic surgery would have helped clarify the results; however, there were not enough studies with consistent dosing regimens or surgical categories to support this analysis. In addition, variability in study design and reporting quality introduced potential bias that may have affected the pooled outcomes. Finally, all included studies were published in English and conducted in different healthcare settings, which may affect the broader applicability of the findings.

## Conclusion

Gabapentinoids, including gabapentin and pregabalin, were associated with statistically significant reductions in postoperative pain scores and opioid consumption following orthopedic surgeries. However, the absolute magnitude of these effects was limited and of uncertain clinical relevance. Additionally, the use of gabapentinoids was linked to an increased incidence of adverse effects, particularly dizziness and visual disturbances. These findings indicate that while gabapentinoids may have a role in select contexts, their routine use for postoperative pain management in orthopedic patients is not strongly supported by the current evidence. Clinical decisions should be individualized, taking into account the potential for side effects, patient-specific risk factors, and the type of surgical procedure. Further research is needed to better define optimal dosing, timing of administration, and patient populations that may derive greater benefit.

## Supplementary Information

Supplementary Material 1.

## Data Availability

No datasets were generated or analysed during the current study.
